# The prognosis of TP53 and EGFR co-mutation in patients with advanced lung adenocarcinoma and intracranial metastasis treated with EGFR-TKIs

**DOI:** 10.3389/fonc.2023.1288468

**Published:** 2024-02-05

**Authors:** Weiguo Gu, Penghui Liu, Jiaming Tang, Jianfei Lai, Siya Wang, Jiaming Zhang, Jinbiao Xu, Jianxiong Deng, Feng Yu, Chao Shi, Feng Qiu

**Affiliations:** ^1^ Department of Oncology, The First Affiliated Hospital, Jiangxi Medical College, Nanchang University, Nanchang, Jiangxi, China; ^2^ Departerment of Oncology, Gaoxin Branch of the First Affiliated Hospital of Nanchang University, Nanchang, Jiangxi, China; ^3^ Nanchang Key Laboratory of Tumor Gene Diagnosis and Innovative Treatment Research, Gaoxin Branch of the First Affiliated Hospital of Nanchang University, Nanchang, Jiangxi, China

**Keywords:** lung cancer, brain metastasis, EGFR, TP53, prognosis

## Abstract

**Background:**

TP53 mutation is a poor factor for non-small cell lung cancer (NSCLC), while the effect of TP53 on prognosis in epidermal growth factor receptor (EGFR)-mutated lung adenocarcinoma (LUAD) with brain metastasis remains elusive and needs further exploration.

**Methods:**

We retrospectively analyzed 236 patients and tested for TP53- and EGFR-mutant status in metastasis LUAD patients who had received first-line EGFR-tyrosine kinase inhibitor (TKI) treatment. Survival rates were calculated by the Kaplan–Meier method. Furthermore, univariate and multivariate Cox analyses were performed to identify the independent prognostic factors.

**Results:**

There were 114 patients with confirmed non-brain metastasis (NBM), 74 patients with preliminary diagnosis early brain metastasis (EBM), and 48 patients with late brain metastasis (LBM). TP53 and EGFR co-mutations were found in 35/236 patients (14.8%). The median progression-free survival (PFS) and overall survival (OS) in the EGFR mutation and TP53 wild-type group were significantly longer than those in the EGFR and TP53 co-mutation group in all advanced LUAD or NBM. Concurrently, PFS and OS were found to be not significant in EBM and LBM patients. Subgroup analysis revealed longer median PFS and OS in the TP53 wild-type group compared to the TP53 mutant group in L858R patients and not significant in EGFR Exon 19 deletion patients. In LBM patients, the time to brain metastasis in the EGFR mutation and TP53 wild-type group was longer than that in the EGFR and TP53 co-mutation group, and TP53 mutant status was an independent prognostic factor for brain metastasis. The TP53 wild-type group exhibited a higher objective remission rate (ORR) and disease control rate (DCR) than the TP53 mutant group in NBM, EBM, and LBM patients, irrespective of primary lung and brain metastatic lesions.

**Conclusion:**

TP53/EGFR co-mutation patients receiving first-line EGFR-TKI treatment had poor prognoses in advanced LUAD, especially with L858R mutation. Moreover, TP53/EGFR co-mutation patients treated with EGFR-TKIs may more easy developed intracranial metastasis.

## 1 Introduction

Lung cancer remains the high incident cases in worldwide (1); lung adenocarcinoma (LUAD) accounts for 40%–50% of non-small cell lung cancer (NSCLC) (2). For early-stage NSCLC, the 5-year survival rate is more than 70% (3). However, there is a lack of awareness about routine physical examination in backward areas, and some individuals have no specific symptoms of peripheral LUAD; nearly 50%–60% of patients found distant metastasis at their first diagnosis (4). Nearly 30%–50% of the NSCLC patients progressed to brain metastasis following the preliminary diagnosis or after the anti-tumor treatment (5); even with whole brain radiation therapy (WBRT), stereotactic radiation therapy (SRT), and postoperation or epidermal growth factor receptor tyrosine kinase inhibitors (EGFR-TKIs), many patients still have a poor prognosis (4–7). The cellular and molecular mechanisms of brain metastasis were need further studied; however, the relationship between oncogenic driver mutations and LUAD of brain metastasis remains undefined (8).

EGFR-TKIs are the first- or further-line standard treatment in advanced NSCLC with EGFR mutation, and overall survival (OS) of such patients prolongs from 1 year to 20–30 months (9, 10). However, some patients develop brain or leptomeningeal metastases, having a median OS of 12 months or worse (11, 12). The studies showed that the patients of EGFR T790M-mutant NSCLC with central nervous system (CNS) metastases progressed on first- or second-generation EGFR-TKI and after osimertinib therapy had a high disease control rate (DCR) and objective remission rate (ORR) (13), and prolonged progression-free survival (PFS) (14, 15), since osimertinib has greater penetration of the blood–brain barrier and higher brain exposure compared with other EGFR-TKIs (16). Moreover, WBRT and chemotherapy are potential crucial treatment strategies for EGFR-wild type advanced NSCLC with CNS (17, 18).

TP53/EGFR co-mutations are present in nearly 17%–70% of advanced NSCLC, affecting cancer cell and non-cell-autonomous cancer features, resulting in genomic instability (19, 20). Li et al. reported that the median PFS and OS in TP53 wild type were the longest compared to exon 4 or 7 of TP53 and other TP53 mutations (*p* < 0.05), indicating TP53 as a promising predictive and prognostic indicator in EGFR-mutated advanced NSCLC on EGFR-TKIs (21). The study also demonstrated that TP53/EGFR co-mutations had lower response rates and shorter PFS than TP53 wild type in NSCLC with EGFR-TKI therapy; TP53 status did not impact the probability of developing CNS metastases either from diagnosis or from the start of TKIs at 5 years; however, this study included stages I–III (22). Jiao et al. suggested that the TP53 wild-type group had a better prognosis than the TP53 mutant group in EGFR wild type (23).

Many studies have stated that TP53 and EGFR co-mutation impact prediction and prognosis in advanced LUAD patients treated with EGFR-TKIs, while the effect on brain metastasis remains elusive and needs further investigation. Therefore, we performed a retrospective study to analyze the prognostic usefulness of TP53 and EGFR co-mutation in advanced LUAD with brain metastasis and EGFR-TKIs as the first-line treatment. It is critical to elucidate the predictive and prognostic TP53-mutated status in EGFR-mutated advanced LUAD patients with brain metastasis, treated with first-line EGFR-TKIs, and explore new molecular biomarkers and treatment approaches.

## 2 Patients and methods

### 2.1 Patients and data collection

We retrospectively identified patients with histologically proven TP53 and EGFR mutation status in metastasis LUAD and received EGFR-TKIs as first-line therapy between January 2014 and July 2021. The following patient characteristics were included: smoking index (400), TNM stages, EGFR and TP53-mutated status, gender, age, and blood biochemical indicators. This study included the following: (1) patients with TP53 and EGFR mutant status tested in metastasis LUAD with or without brain metastasis who had received EGFR-TKIs, in conjunction with WBRT or chemotherapy, and patients with data integrity and evaluable target lesions; (2) patients with LUAD without other malignant tumors; and (3) patients who have complete clinical data.

### 2.2 Post-treatment evaluation

The post-treatment evaluation included computed tomography (CT), bone scan, and craniocerebral nuclear magnetic resonance imaging (MRI). The short-term effects were evaluated by Response Evaluation Criteria in Solid Tumors 1.1 (RECIST1.1). This study of primary endpoint was mainly OS and PFS, which was measured from the starting date of first-line treatment. The LUAD with non-brain metastasis (NBM) means patients having no brain metastasis at preliminary or late diagnosis; early brain metastasis (EBM) means patients with brain metastasis at preliminary diagnosis and mainly received third-generation TKIs; late brain metastasis (LBM) refers to patients with brain metastasis. The patients received osimertinib for EBM or with T790M mutation. Moreover, a portion of patients received EGFR-TKIs combined with chemotherapy (platinum-based combination chemotherapy) in EGFR Exon 19 deletion or L858R mutation with resistant gene (such as her2, KRAS, met, etc.). Follow-up methods included short messages, outpatient visits, telephone conversations, and regular reviews. Furthermore, the follow-up time of the cutoff date was 30 December 2022, and the median follow-up period was 32 months.

### 2.3 Statistical analysis

Chi-square or Fisher’s exact test was used for categorical variables. Multivariate logistic regression analysis to found the independent risk factors (24). The Kaplan–Meier method was employed to calculate survival rates. Log-rank test was used for univariate prognostic analysis, and Cox proportional hazards models were used for multivariate prognostic analysis to identify independent prognostic factors (24). In statistical results, *p* < 0.05 was considered significant. Statistical analysis was performed using IBM SPSS 22 and GraphPad Prism version 8.0. The mean value of peripheral serum routine blood test and tumor biomarkers of normal value were taken as cutoff points for statistical analysis.

## 3 Results

### 3.1 Correlation between LUAD brain metastasis and NBM in clinicopathological characteristics

**Figure 1:**
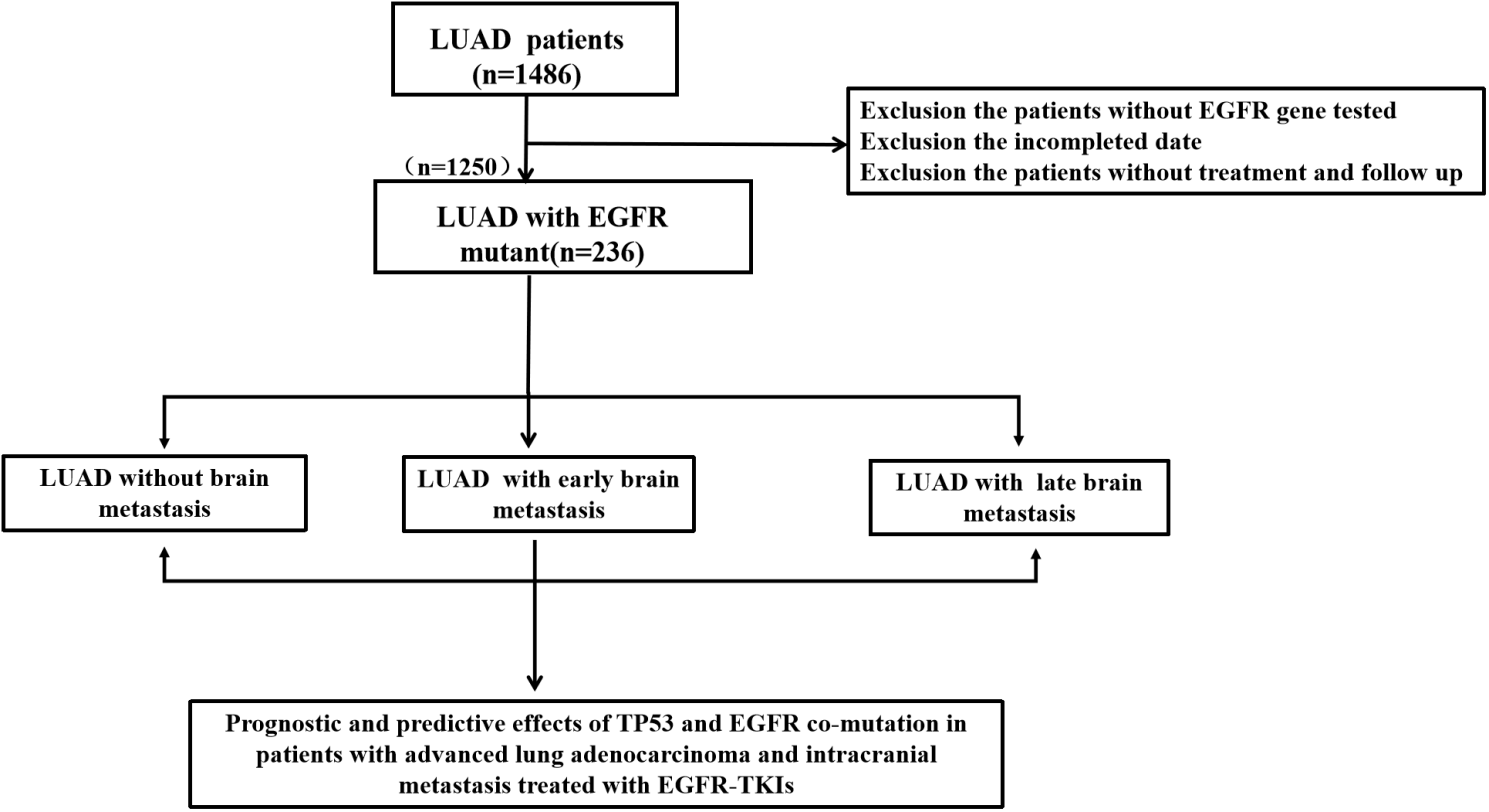
A flowchart for the study to enrolled the patients.

**Figure 2:**
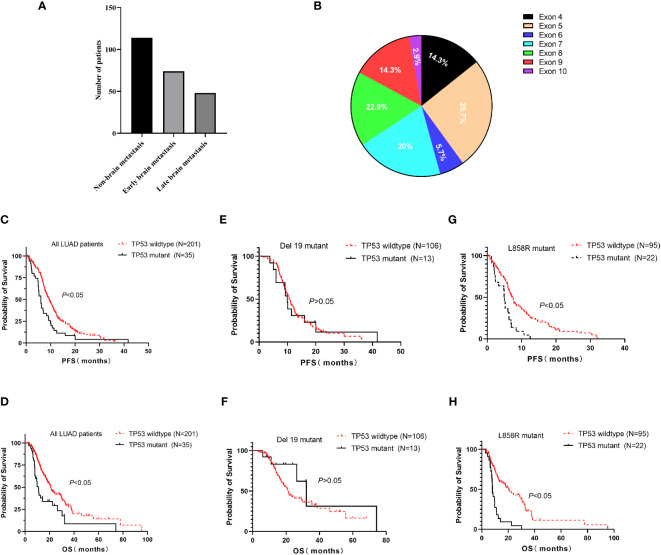
The relation between TP53 and EGFR-TKI therapy in EGFR-mutant advanced LUAD. **(A)** The number of LUAD patients in NBM, EBM, and LBM. **(B)** Pie chart about TP53 missense mutant status. **(C, D)** The TP53 wild-type group had a high median PFS and OS than the TP53 mutant group in all advanced LUAD patients. **(E, F)** The TP53 mutant group had no effect on PFS and OS compared to the TP53 wild-type group in EGFR Exon 19 deletion patients. **(G, H)** Subgroup analysis revealed longer median PFS and OS in the TP53 wild-type group compared to the TP53 mutant group in L858R patients. EBM, early brain metastasis; LBM, late brain metastasis; and NBM, non-brain metastasis.

We performed a retrospective examination of 236 patients who were evaluated for TP53- and EGFR-mutant status and received first-line EGFR-TKIs for advanced or postoperatively recurrent LUAD. A flowchart for the study is shown in **Figure 1**. There were 114 patients with confirmed NBM, 74 with preliminary diagnosis EBM, and 48 with LBM (**Figure 2A**). TP53 and EGFR co-mutations were found in 35/236 (14.8%) patients; these TP53-mutant types belonged to missense patients, and nearly 10 non-missense patients were excluded; the different TP53 mutant statuses were as follows: Exon 4 mutation were 5 patients, Exon 5 mutation were 9 patients, Exon 6 mutation were 2 patients, Exon 7 mutation were 7 patients, Exon 8 mutation were 6 patients, Exon 9 mutation were 5 patients , Exon 10 mutation were 1 patients (**Figure 2B**). Moreover, EGFR exon 19 deletion was found in 119 patients, and EGFR L858R was found in 117 patients. There were 25 patients with T790M mutation (18 patients with EGFR exon 19 deletion and T790M mutation and 7 patients with EGFR L858R and T790M co-mutation). The univariate logistic analysis showed a significant relationship between age, performance status (PS), M stages, TNM stages, platelet-to-lymphocyte ratio (PLR) ratio, neutrophil-to-lymphocyte ratio (NLR) ratio, and serum cyfra211 and EBM in LUAD (*p* < 0.05); age and EGFR mutant were significantly related to LBM (**Table 1**). Multivariate logistic regression analysis revealed that age, ECOG PS, and M stages were independent risk factors associated with EBM, and age was an independent risk factor for LBM (**Table 2**).

**Table 1:** Characteristics of 236 patients who received first-line therapy in LUAD with EGFR mutation.

Characteristic		NBM	LUAD-EBM			LUAD-LBM		
		*N*	*N*	OR (95% CI)	*p*	*N*	OR (95% CI)	*p*
**Gender**	Male	59	32	Ref		22	Ref	
	Female	55	42	1.408 (0.782–2.536)	0.255	26	1.286 (0.645–2.493)	0.492
**Age (years)**	≤60	50	47	Ref		32	Ref	
	>60	64	27	0.449 (0.246–0.818)	0.009	16	0.391 (0.193–0.791)	0.009
**ECOG PS**	0–1	84	39	Ref		35	Ref	
	≥2	30	35	2.513 (1.354–4.662)	0.003	13	1.04 (0.486–2.226)	0.92
**Smoking index^a^**	<400	82	54	Ref		30	Ref	
	≥400	32	20	0.949 (0.493–1.829)	0.876	18	1.538 (0.754–3.127)	0.237
**T stages**	T1	28	8	Ref	1.141	12	Ref	0.612
	T2	21	15	2.5 (0.894–6.987)	0.081	10	1.111 (0.404–3.057)	0.838
	T3	6	6	3.5 (0.883–13.879)	0.075	5	1.944 (0.496–7.621)	0.34
	T4	59	45	2.669 (1.111–6.412)	0.028	21	0.831 (0.359–1.923)	0.665
**N stages**	N0	34	12	Ref	0.055	11	Ref	0.628
	N1	2	0	0.00 (0.000–0.001)	0.999	2	3.091 (0.388–24.606)	0.286
	N2	66	45	1.932 (0.904–4.128)	0.089	31	1.452 (0.651–3.240)	0.363
	N3	12	17	4.014 (1.492–10.797)	0.006	4	1.03 (0.275–3.857)	0.965
**M stages**	M0	33	4	Ref		21	Ref	
	M1	81	70	7.13 (2.407–21.118)	<0.001	27	0.524 (0.26–1.054)	0.07
**TNM stages**	Recurrent	25	2	Ref	0.003	14	Ref	0.334
	IIIB–C	7	2	3.571 (0.424–30.102)	0.242	5	1.276 (0.34–4.78)	0.718
	IV	82	70	10.671 (2.441–46.65)	0.002	29	0.632 (0.29–1.377)	0.248
**TP53 status**	Wild type	96	64	Ref		41	Ref	
	Mutant	18	10	0.833 (0.361–1.921)	0.669	7	0.911 (0.353–2.346)	0.846
**PLR ratio^b^**	≤184	71	31	Ref		30	Ref	
	>184	43	43	2.29 (1.26–4.162)	0.007	18	0.991 (0.494–1.988)	0.979
**NLR ratio^b^**	≤3.68	80	40	Ref		35	Ref	
	>3.68	34	34	2.0 (1.089–3.675)	0.026	13	0.874 (0.412–1.855)	0.726
**NSE (ng/mL)^c^**	≤16.3	47	31	Ref		20	Ref	
	>16.3	67	43	0.973 (0.537–1.762)	0.928	28	0.947 (0.476–1.884)	0.877
**CEA (ng/mL)^c^**	≤6.5	42	19	Ref		17	Ref	
	>6.5	72	55	1.689 (0.885–3.22)	0.112	30	1.029 (0.508–2.086)	0.936
**Cyfra211 (ng/mL)^c^**	≤3.3	49	21	Ref		25	Ref	
	>3.3	65	53	1.903 (1.017–3.561)	0.044	23	0.694 (0.352–1.365)	0.289
**Ki-67 expression**	<60	46	22	Ref		25	Ref	
	≥60	68	52	1.599 (0.857–2.982)	0.14	23	0.316–1.227	0.171
**EGFR mutant**	Exon 19 deletion	62	38	Ref		19	Ref	
	Exon 21 L858R	52	36	1.13 (0.629–2.03)	0.684	29	2.49 (1.23–5.042)	0.011

ECOG PS, Eastern Cooperative Oncology Group performance status; EGFR, epidermal growth factor receptor; PLR, platelet-to-lymphocyte ratio; NLR, neutrophil-to-lymphocyte ratio; CEA, carcinoembryonic antigen; Cyfra211, cytokeratin 19 fragment; Ki-67, nuclear proliferation antigen 67; Ref, reference; LUAD, lung adenocarcinoma; EBM, early brain metastasis; LBM, late brain metastasis. a = number of cigarettes per day × smoking age; b = the cutoff points were used as the mean value; c = the cutoff points were used for relevant assay kits.

**Table 2:** Multivariate logistic proportional hazards regression analysis in advanced LUAD.

Characteristic	Groups	LUAD-EBM		LUAD-LBM	
		OR (95% CI)	*p*	OR (95% CI)	*p*
**Age (years)**	≤60	Ref		Ref	
	>60	0.449 (0.238–0.848)	0.014	0.385 (0.187–0.793)	0.01
**ECOG PS**	0–1	Ref			
	≥2	1.934 (1.005–3.72)	0.048		
**M stages**	M0	Ref			
	M1	6.235 (2.056–18.913)	0.001		

ECOG PS, Eastern Cooperative Oncology Group performance status; LUAD, lung adenocarcinoma; Ref, reference; EBM, early brain metastasis; LBM, late brain metastasis.

### 3.2 Univariate and multivariate survival analyses of TP53 and EGFR co-mutation in advanced LUAD with brain metastasis

Numerous studies demonstrated a connection between TP53 mutation and poor prognosis. Therefore, we analyzed the effect of EGFR-TKIs as the first-line treatment in advanced LUAD. In all advanced LUAD patients, the TP53 wild-type group had a high median PFS (9.4 vs. 6 months, *p* < 0.05, **Figure 2C**) and OS (20.8 vs. 10.9 months, *p* < 0.05, **Figure 2D**) compared to the TP53 mutant group. In EGFR Exon 19 deletion patients, the TP53 mutant group exhibited no effect on PFS (10.6 vs. 9.9 months, *p* > 0.05, **Figure 2E**) and OS (32.2 vs. 21.3 months, *p* > 0.05, **Figure 2F**) compared to the TP53 wild-type group. In L858R patients, the TP53 wild-type group displayed an effect on median PFS (7.2 vs. 4.9 months, *p* < 0.05, **Figure 2G**) and OS (20.4 vs. 8.0 months, *p* < 0.05, **Figure 2H**) compared to the TP53 mutant group.

**Figure 3:**
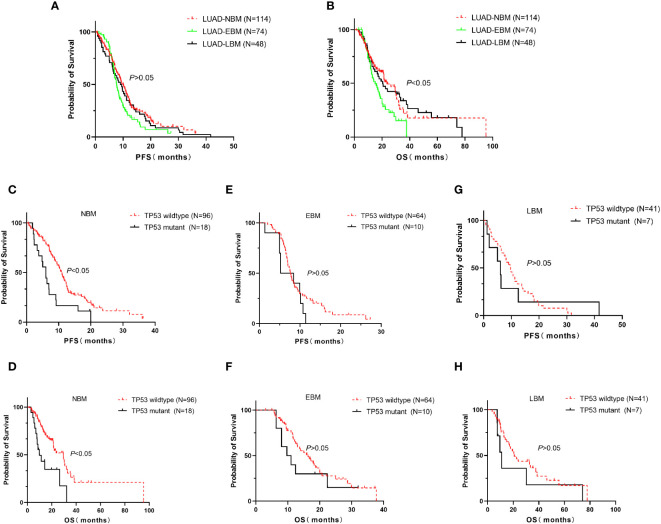
Correlation between TP53 mutation and brain metastasis in advanced LUAD first line treated with EGFR-TKIs. In all advanced LUAD patients, the median of PFS in the EBM group was insignificant compared to the NBM and LBM groups **(A)**, while the median of OS in the NBM group was the longest among all groups **(B)**. **(C, D)** The TP53 wild-type group had a higher median PFS and OS than the TP53 mutant group in NBM patients. **(E–H)** In EBM and LBM patients, the TP53 wild-type group did not affect PFS and OS compared to the TP53 mutant group. EBM, early brain metastasis; LBM, late brain metastasis; and NBM, non-brain metastasis.

We further investigated the relationship between TP53 mutant and brain metastasis in advanced LUAD treated with EGFR-TKIs. The median of PFS (7.7 vs. 10 vs. 9 months, *p* > 0.05, **Figure 3A**) in the EBM group was not significant compared to the NBM and LBM groups, while the median OS (15 vs. 23.5 vs. 20.4 months, *p* < 0.05, **Figure 3B**) in the EBM group was the shortest among all groups. Furthermore, the TP53 wild-type group had a higher median PFS (11 vs. 6 months, *p* < 0.05, **Figure 3C**) and OS (29.4 vs. 9.8 months, *p* < 0.05, **Figure 3D**) than the TP53 mutant group in NBM patients. In EBM and LBM patients, the TP53 wild-type group did not impact PFS and OS compared to the TP53 mutant group (*p* > 0.05, **Figures 3E–H**).

Additional evaluation of the association between clinicopathological information and prognosis in EBM and LBM patients was carried out. The univariate Cox analysis showed that gender, Ki-67 expression, and smoking index correlated with PFS in EBM patients; TNM stages and Ki-67 expression were associated with PFS in LBM patients. Multivariate Cox analysis (univariate analysis *p* < 0.05) showed that smoking index and Ki-67 expression were independent prognostic factors for PFS (*p* < 0.05) in EBM patients; TNM stages and Ki-67 expression were also independent prognostic factors for PFS (*p* < 0.05) in LBM patients (**Table 3**). Moreover, the univariate Cox analysis showed that gender and smoking index were associated with OS in EBM patients; TNM stages and Ki-67 expression were associated with OS in LBM patients. Multivariate Cox analysis (univariate analysis *p* < 0.05) showed that gender was an independent prognostic factor for OS in EBM patients (*p* < 0.05); TNM stages and Ki-67 expression were also independent prognostic factors for OS in LBM patients (*p* < 0.05) (**Table 4**).

**Table 3:** Univariate and multivariate COX analysis of brain metastasis for PFS in LUAD.

Characteristic		LUAD-EBM				LUAD-LBM			
		Univariate		Multivariate		Univariate		Multivariate	
		HR (95% CI)	*p*	HR (95% CI)	*p*	HR (95% CI)	*p*	HR (95% CI)	*p*
**Gender**	Male	Ref				Ref			
	Female	0.498 (0.296−0.838)	0.009			0.643 (0.348−1.187)	0.158		
**Age (years)**	≤60	Ref				Ref			
	>60	1.148 (0.67−1.967)	0.616			0.846 (0.448−1.598)	0.607		
**ECOG PS**	0–1	Ref				Ref			
	≥2	0.878 (0.524−1.473)	0.622			1.477 (0.773−2.82)	0.238		
**Smoking index**	<400	Ref		Ref		Ref			
	≥400	2.551 (1.433−4.541)	0.001	2.492 (1.383−4.49)	0.002	1.53 (0.815−2.872)	0.186		
**T stages**	T1	Ref	0.325			Ref	0.688		
	T2	0.5 (0.203−1.231)	0.132			0.717 (0.30−1.716)	0.455		
	T3	1.09 (0.354−3.361)	0.88			1.363 (0.475−3.905)	0.565		
	T4	0.626 (0.287−1.366)	0.239			1.054 (0.513−2.167)	0.885		
**N stages**	N0	Ref	0.341			Ref	0.082		
	N1	–	–			1.281 (0.268−6.13)	0.756		
	N2	0.929 (0.445−1.938)	0.844			2.713 (1.232−5.977)	0.013		
	N3	0.587 (0.244−1.412)	0.234			2.494 (0.738−8.426)	0.141		
**M stages**	M0	–	–			Ref			
	M1	–	–			0.918 (0.511−1.647)	0.773		
**TNM stages**	Recurrent	Ref	0.836			Ref	0.012	Ref	0.003
	IIIB–C	0.785 (0.108−5.694)	0.811			4.876 (1.601−14.85)	0.005	6.784 (2.163−21.27)	0.001
	IV	1.182 (0.286−4.875)	0.817			1.07 (0.551−2.076)	0.842	1.209 (0.619−2.36)	0.579
**TP53 status**	Wild type	Ref				Ref			
	Mutant	1.587 (0.793−3.176)	0.192			1.029 (0.427−2.48)	0.949		
**PLR ratio**	≤184	Ref				Ref			
	>184	1.377 (0.822−2.309)	0.224			1.16 (0.625−2.154)	0.638		
**NLR ratio**	≤3.68	Ref				Ref			
	>3.68	1.133 (0.678−1.893)	0.633			1.435 (0.736−2.798)	0.289		
**NSE (ng/mL)**	≤16.3	Ref				Ref			
	>16.3	0.866 (0.509−1.474)	0.597			1.168 (0.642−2.123)	0.611		
**CEA (ng/mL)**	≤6.5	Ref				Ref			
	>6.5	1.09 (0.597−1.989)	0.78			1.479 (0.779−2.811)	0.232		
**Cyfra211 (ng/mL)**	≤3.3	Ref				Ref			
	>3.3	1.289 (0.737−2.254)	0.373			1.435 (0.786−2.619)	0.239		
**Ki-67 expression**	<60	Ref		Ref		Ref			
	≥60	1.973 (1.087−3.579)	0.025	2.112 (1.155−3.861)	0.015	1.989 (1.083−3.652)	0.027	2.299 (1.236−4.277)	0.009

ECOG PS, Eastern Cooperative Oncology Group performance status; PLR, platelet-to-lymphocyte ratio; NLR, neutrophil-to-lymphocyte ratio; CEA, carcinoembryonic antigen; Cyfra211, cytokeratin 19 fragment; Ki-67, nuclear proliferation antigen 67; Ref, reference; LUAD, lung adenocarcinoma; EBM, early brain metastasis; LBM, late brain metastasis.

**Table 4:** Univariate and multivariate COX analysis of brain metastasis for OS in LUAD.

Characteristic		LUAD-EBM				LUAD-LBM			
		Univariate		Multivariate		Univariate		Multivariate	
		HR (95% CI)	*p*	HR (95% CI)	*p*	HR (95% CI)	*p*	HR (95% CI)	*p*
**Gender**	Male	Ref		Ref		Ref			
	Female	0.364 (0.2–0.664)	0.001	0.343 (0.184–0.637)	0.001	0.878 (0.44–0.1.745)	0.771		
**Age (years)**	≤60	Ref				Ref			
	>60	1.0 (0.543–1.841)	0.999			1.314 (0.646–2.675)	0.451		
**ECOG PS**	0–1	Ref				Ref			
	2–3	0.786 (0.437–1.413)	0.42			1.602 (0.731–3.51)	0.239		
**Smoking index**	<400	Ref				Ref			
	≥400	3.25 (1.73–6.106)	<0.001			1.309 (0.649–2.637)	0.452		
**T stages**	T1	Ref	0.468			Ref	0.389		
	T2	0.667 (0.245–1.814)	0.427			0.443 (0.144–1.365)	0.156		
	T3	1.62 (0.449–5.837)	0.461			1.32 (0.4–4.359)	0.648		
	T4	0.774 (0.315–1.898)	0.575			0.805 (0.351–1.848)	0.609		
N **stages**	N0	Ref	0.207			Ref	0.489		
	N1	1.701 (0.661–4.376)	0.271			0.862 (0.179–4.144)	0.853		
	N2	0.905 (0.295–2.779)	0.862			1.412 (0.61–3.265)	0.42		
	N3	–	–			0.481 (0.1–2.312)	0.361		
M **stages**	M0	Ref				Ref			
	M1	1.064 (0.328–3.454)	0.918			0.922 (0.457–1.862)	0.821		
**TNM stages**	Recurrent	Ref	0.749			Ref	0.001	Ref	<0.001
	IIIB–C	2.532 (0.228–28.09)	0.449			9.949 (2.634–37.57)	0.001	17.68 (4.37–71.47)	<0.001
	IV	1.784 (0.244–13.04)	0.568			0.878 (0.386–1.996)	0.755	0.87 (0.38–2.004)	0.874
**TP53 status**	Wild type	Ref				Ref			
	Mutant	1.462 (0.68–3.144)	0.331			1.661 (0.67–4.117)	0.273		
**PLR ratio**	≤184	Ref				Ref			
	>184	1.06 (0.593–1.896)	0.843			1.673 (0.851–3.291)	0.136		
**NLR ratio**	≤3.68	Ref				Ref			
	>3.68	0.94 (0.523–1.688)	0.836			1.392 (0.675–2.872)	0.37		
**NSE (ng/mL)**	≤16.3	Ref				Ref			
	>16.3	0.821 (0.457–1.475)	0.509			1.001 (0.492–2.036)	0.999		
**CEA (ng/mL)**	≤6.5	Ref				Ref			
	>6.5	1.0 (0.502–2.013)	0.989			1.342 (0.634–2.839)	0.442		
**Cyfra211 (ng/mL)**	≤3.3	Ref				Ref			
	>3.3	1.486 (0.769–2.871)	0.239			1.409 (0.714–2.778)	0.323		
**Ki-67 expression**	<60	Ref				Ref		Ref	
	≥60	1.183 (0.622–2.25)	0.608			2.137 (1.058–4.319)	0.034	3.017 (1.418–6.42)	0.004

ECOG PS, Eastern Cooperative Oncology Group performance status; PLR, platelet-to-lymphocyte ratio; NLR, neutrophil-to-lymphocyte ratio; CEA, carcinoembryonic antigen; Cyfra211, cytokeratin 19 fragment; Ki-67, nuclear proliferation antigen 67; Ref, reference; LUAD, lung adenocarcinoma; EBM, early brain metastasis; LBM, late brain metastasis.

### 3.3 Univariate and multivariate Cox analyses of EGFR and TP53 co-mutation status and time to brain metastasis in advanced LUAD

**Figure 4:**
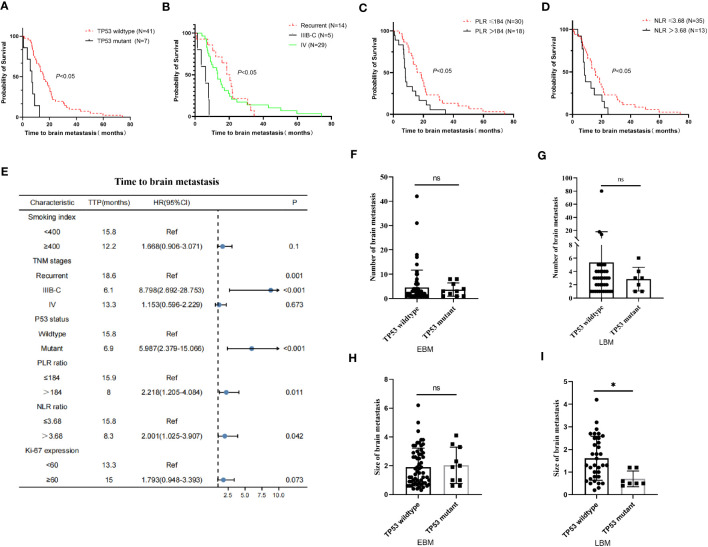
Relationship between clinical–pathological features and time to brain metastasis in EGFR-mutant advanced lung adenocarcinoma with EGFR-TKI therapy. **(A)** The TP53 wild-type group exhibited a significantly longer time to brain metastasis than the TP53 mutant group. **(B)** The IIIB–C group had a significantly shorter time to brain metastasis than the local recurrence and IV stage group. **(C, D)** The low PLR and NLR groups took significantly longer time to brain metastasis than the high PLR and NLR groups. **(E)** The univariate Cox analysis showed that gender, smoking index, and Ki-67 expression were correlated with time to brain metastasis in advanced lung adenocarcinoma (*p* < 0.05); these factors were drawn using a forest map. **(F, G)** The number of brain metastasis in the TP53 wild-type group was insignificant compared to the TP53 mutant group in EBM and LBM patients. The diameter of brain metastasis in the TP53 wild-type group was insignificant compared to the TP53 mutant group in EBM **(H)**, while the TP53 wild-type group was greater than the TP53 mutant group in LBM **(I)**.

The TP53 wild-type group had a longer OS than the TP53 mutant group in all LUAD, NBM, or L858R patients; therefore, we further analyzed the correlation between EGFR and TP53 co-mutation status and time to brain metastasis in LUAD patients. The TP53 wild-type group had a significantly longer time (15.8 vs. 6.9 months, *p* < 0.05, **Figure 4A**) to brain metastasis than the TP53 mutant group; the IIIB–C stage group had a significantly shorter time (6.1 vs. 18.6 vs. 13.3 months, *p* < 0.05, **Figure 4B**) to brain metastasis than the local recurrence and IV stage groups; the low PLR group had a significantly longer time (15.9 vs. 8 months, *p* < 0.05, **Figure 4C**) to brain metastasis than the high PLR group; the low NLR group had a significantly longer time (15.8 vs. 8.3 months, *p* < 0.05, **Figure 4D**) to brain metastasis than the high NLR group. Moreover, univariate and multivariate Cox analysis showed that TNM stages and TP53 mutant status were independent prognostic factors for time to brain metastasis in LUAD with LBM (**Table 5**; **Figure 4E**).

**Table 5:** Univariate and multivariate COX analysis of time to brain metastasis in LUAD.

Characteristic		TTP (months)	LUAD-EBM			
			Univariate		Multivariate	
			HR (95% CI)	*p*	HR (95% CI)	*p*
**Gender**	Male	**13**	Ref			
	Female	13.3	0.793 (0.444–1.416)	0.432		
**Age (years)**	≤60	**13.3**	Ref			
	>60	13	1.071 (0.579–1.98)	0.827		
**ECOG PS**	0–1	**15**	Ref			
	2–3	13.3	1.551 (0.803–2.993)	0.191		
**Smoking index**	<400	**15.8**	Ref			
	≥400	12.2	1.668 (0.906–3.071)	0.1		
**T stages**	T1	**18.6**	Ref	0.342		
	T2	17.2	0.741 (0.308–1.783)	0.503		
	T3	8.3	1.11 (0.372–3.311)	0.851		
	T4	12.7	1.509 (0.737–3.09)	0.261		
**N stages**	N0	13	Ref	0.231		
	N1	17.2	1.072 (0.227–5.07)	0.93		
	N2	13.3	1.72 (0.798–3.711)	0.167		
	N3	6.9	0.518 (0.142–1.885)	0.318		
**M stages**	M0	18.6	Ref			
	M1	13	1.261 (0.703–2.261)	0.437		
**TNM stages**	Recurrent	18.6	Ref	0.001	Ref	<0.001
	IIIB–C	6.1	8.798 (2.692–28.753)	<0.001	10.198 (2.921–35.609)	<0.001
	IV	13.3	1.153 (0.596–2.229)	0.673	0.95 (0.477–1.892)	0.884
**TP53 status**	Wildtype	15.8	Ref		Ref	
	Mutant	6.9	5.987 (2.379–15.066)	<0.001	7.458 (2.783–19.986)	<0.001
**PLR ratio**	≤184	15.9	Ref			
	>184	8.0	2.218 (1.205–4.084)	0.011		
**NLR ratio**	≤3.68	15.8	Ref			
	>3.68	8.3	2.001 (1.025–3.907)	0.042		
**NSE (ng/mL)**	≤16.3	13	Ref			
	>16.3	15.8	0.908 (0.502–1.643)	0.75		
**CEA (ng/mL)**	≤6.5	13.3	Ref			
	>6.5	13.3	1.275 (0.682–2.383)	0.447		
**Cyfra211 (ng/mL)**	≤3.3	18.6	Ref			
	>3.3	9.1	1.382 (0.772–2.475)	0.276		
**Ki-67 expression**	<60	13.3	Ref			
	≥60	15	1.793 (0.948–3.393)	0.073		

ECOG PS, Eastern Cooperative Oncology Group performance status; PLR, platelet-to-lymphocyte ratio; NLR, neutrophil-to-lymphocyte ratio; CEA, carcinoembryonic antigen; Cyfra211, cytokeratin 19 fragment; Ki-67, nuclear proliferation antigen 67; Ref, reference; LUAD, lung adenocarcinoma; EBM, early brain metastasis; LBM, late brain metastasis; TTP, time to brain progression.

Moreover, we further analyzed the relationship between TP53 mutant status and number size of brain metastasis. In EBM and LBM patients, the number of brain metastasis in the TP53 wild-type group was insignificant compared to the TP53 mutant group (*p* > 0.05, **Figures 4F, G**). The diameter of brain metastasis in the TP53 wild-type group was not significant compared to the TP53 mutant group in EBM (*p* > 0.05, **Figure 4H**), while that of the TP53 wild-type group was greater than the TP53 mutant group in LBM (*p* < 0.05, **Figure 4I**).

### 3.4 Short-term therapeutic effects and TP53 expressions in EBM and LBM

**Figure 5:**
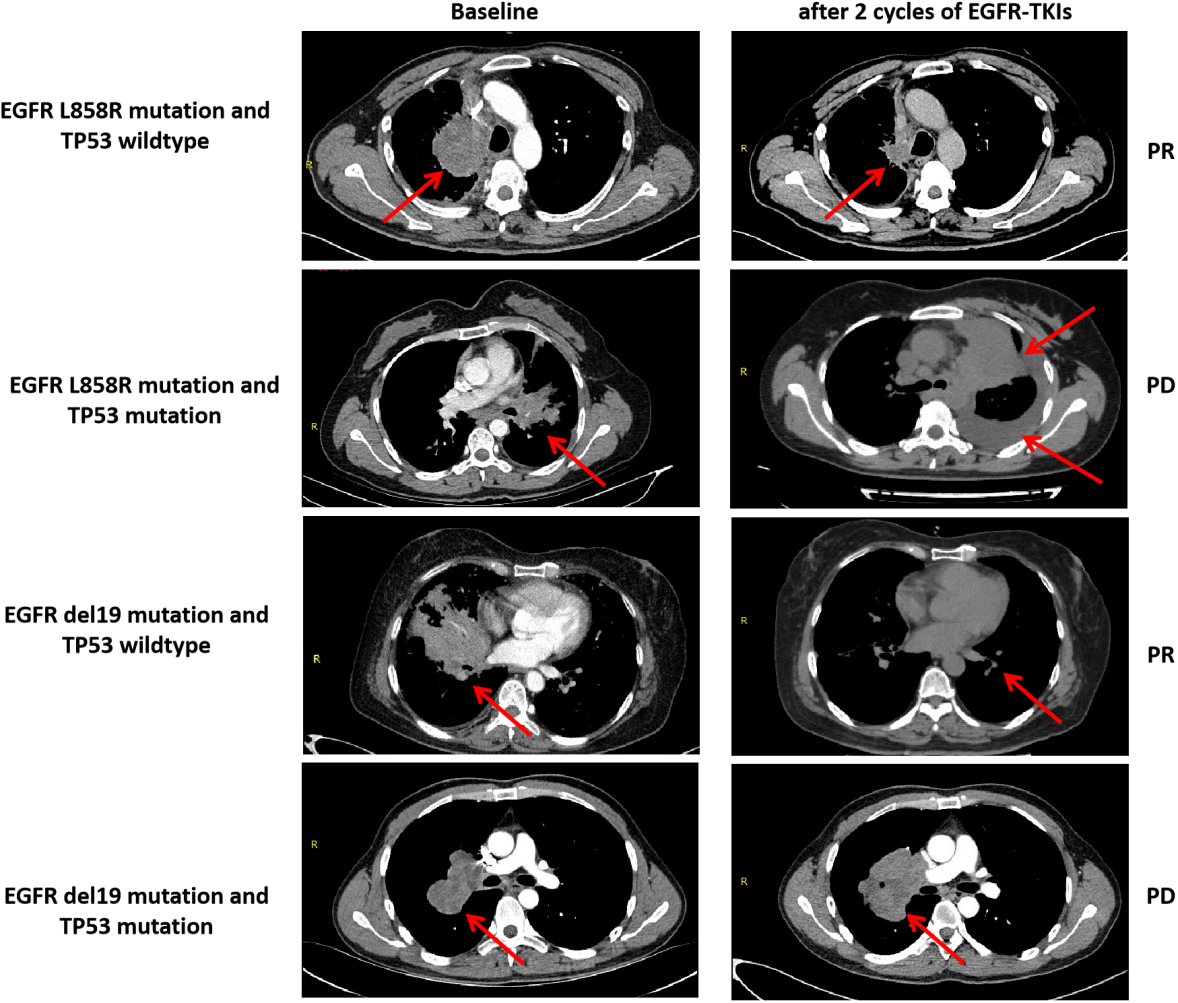
Anti-tumor activity and TP53 mutant status and EGFR-TKIs in four patients with EGFR-mutant advanced LUAD. PR, partial response; PD, progressive disease.

Disease progression was evaluated through imaging examination every 2 months after 3–4 weeks of treatment. In NMB, EBM, and LBM patients with all tumor lesions, the TP53 wild-type group had a higher DCR than the TP53 mutant expression group (*p* < 0.05), but not ORR (*p* > 0.05) (**Table 6**, **Figure 5**). Additionally, we analyzed the TP53 mutant status and brain metastasis of the lesion. In EBM patients, the TP53 wild-type group had a higher CR rate (3.1% vs. 0%) and DCR (84.4% vs. 50%) than the TP53 mutant expression groups. In LBM patients, the TP53 wild-type group had a higher ORR (14.6% vs. 0%) and DCR (68.3% vs. 14.3%) than the TP53 mutant expression group (**Table 7**).

**Table 6:** The short-term efficacy comparison among TP53 mutant status with first-line therapy in all lesions of LUAD.

RECIST	NBM		EBM		LBM	
	P53 wild type	TP53 mutant	TP53 wild type	TP53 mutant	TP53 wild type	TP53 mutant
**CR**	3 (3.1%)	0 (0%)	0 (0%)	0 (0%)	1 (2.4%)	0 (0%)
**PR**	23 (24%)	4 (22.2%)	27 (42.2%)	4 (40%)	6 (14.6%)	1 (14.2%)
**SD**	51 (53.1%)	6 (33.3%)	25 (39.1%)	1 (10%)	25 (61%)	3 (42.9%)
**PD**	19 (19.8%)	8 (44.4%)	12 (18.7%)	5 (50%)	9 (22%)	3 (42.9%)
**ORR**	26 (27.1%)	4 (22.2%)	27 (42.2%)	4 (40%)	7 (17.1%)	1 (14.2%)
**DCR**	77 (80.2%)	10 (55.6%)	52 (81.3%)	5 (50%)	32 (78.1%)	4 (57.1%)

CR, Complete Response; PR, Partial Response; SD, Stable Disease; PD, Progressive Disease; DCR (disease control rate) = (CR + PR + SD)/total cases * 100%, ORR (objective remission rate) = (CR + PR)/total cases * 100%. LUAD, lung adenocarcinoma; NBM, non-brain metastasis; EBM, early brain metastasis; LBM, late brain metastasis.

**Table 7:** The short-term efficacy comparison among TP53 mutant status in brain metastasis lesion of LUAD with EGFR-TKIs or brain radiotherapy.

RECIST	EBM		LBM	
	TP53 wild type	TP53 mutant	TP53 wild type	TP53 mutant
**CR**	2 (3.1%)	0 (0%)	4 (9.8%)	0 (0%)
**PR**	6 (9.4%)	1 (10%)	2 (4.8%)	0 (0%)
**SD**	46 (71.9%)	4 (40%)	22 (53.6%)	1 (14.3%)
**PD**	10 (1.6%)	5 (50%)	13 (31.7%)	6 (85.7%)
**ORR**	8 (12.5%)	1 (10%)	6 (14.6%)	0 (0%)
**DCR**	54 (84.4%)	5 (50%)	28 (68.3%)	1 (14.3%)

CR, Complete Response; PR, Partial Response; SD, Stable Disease; PD, Progressive Disease; DCR (disease control rate) = (CR + PR + SD)/total cases * 100%, ORR (objective remission rate) = (CR + PR)/total cases * 100%. LUAD, lung adenocarcinoma; EBM, early brain metastasis; LBM, late brain metastasis.

## 4 Discussion

Nearly 30%–50% of lung cancer patients with a preliminary diagnosis or who have had anti-tumor therapy develop brain metastases, and their management and prognosis are poor (5–7). The studies showed that the median OS for the ALK/EGFR+ NSCLC brain metastasis was longer than that of the wild type (19.9 vs. 10.1 months, *p* = 0.028) (25). Osimertinib has greater blood–brain barrier penetration, higher brain exposure, and a good prognosis; hence, it has become an essential treatment for EGFR-mutated NSCLC patients with CNS metastases (13–16). Therefore, it is vital to analyze the correlation between driver gene and brain metastasis and explore a novel treatment strategies for brain metastasis.

TP53/EGFR co-mutation is commonly found in advanced NSCLC; the TP53 wild-type has a good prognosis and correlates with primary and acquired resistance to EGFR-TKIs (19–21). The study showed that nondisruptive TP53 mutations are related to poor survival (17.8 months vs. 28.4 months) compared to TP53 wild-type stage IIIB–IV EGFR-mutated NSCLC patients, and multivariate analyses suggested that nondisruptive TP53 mutations were independent prognostic factors associated with a shorter OS (26). Moreover, the mechanism and effect of TP53 in primary sensitivity and acquired resistance to EGFR-TKIs in NSCLC cells showed that TP53 mutations promote the epithelial-to-mesenchymal transition (EMT), activating EGFR mutations and enhancing resistance to osimertinib in H1975 cells (27). Furthermore, a phase III randomized trial (CTONG 0901) was conducted to analyze the relationship between TP53 and EGFR-TKIs in EGFR-mutated advanced NSCLC; the study found that the TP53 wild-type group had the longest median PFS (9.4 vs. 11.0 vs. 14.5 months, *p* = 0.009) and OS (15.8 vs. 20.0 vs. 26.1 months, *p* = 0.004) compared to exon 4 or 7 of TP53 or other TP53 mutations, indicating that TP53 could be a promising predictive and prognostic factor in EGFR-mutated NSCLC (21).

This study also evaluated the effect of TP53 on PFS and OS in EGFR-mutated advanced LUAD patients receiving EGFR-TKI therapy. The median PFS and OS in the TP53 wild-type group were longer than TP53 mutations in all advanced LUAD, and subgroup analysis revealed the relation between EGFR-mutant types and TP53; the effect of TP53-mutated status did not correlate with survival time in EGFR Exon 19 deletion patients. In contrast, TP53 mutation exhibited a shorter PFS and OS than the TP53 wild type in L858R patients. Moreover, the TP53 wild-type group had a longer DCR and ORR than the TP53 mutation. Therefore, the TP53 and EGFR L858R co-mutation may be an important indicator for the predictive effect of EGFR-TKIs in advanced LUAD patients.

Many studies reported that the median OS in NSCLC patients with brain or leptomeningeal metastases is 12 months or worse (11, 12). Labbé et al. found an insignificant association between TP53-mutant status from diagnosis and from the start of TKIs to developing brain metastases; results might be due to the small sample size of LUAD patients, and many patients were second- or further-line postoperatively relapsed patients receiving the EGFR-TKI therapy; these factors could significantly affect the outcomes (22). Our study retrospectively analyzed 236 patients and tested for TP53-mutated status (201 patients were wild type and 35 were mutated) in EGFR-mutant advanced LUAD patients undergoing EGFR-TKIs as the first-line treatment. The NBM patients in TP53 wild-type groups had a longer PFS and OS than TP53 mutations, while the TP53-mutant status had no effect on survival time in EBM or LBM patients. Moreover, the TP53 wild-type group had a higher CR rate and ORR than TP53 mutant groups in LBM and EBM patients receiving EGFR-TKI alone or in combination with craniocerebral radiotherapy. Notably, we interestingly found that the TP53 wild-type group had a significantly longer time to brain metastasis than the TP53-mutated group in advanced LUAD patients. However, the number of brain metastasis in the TP53 wild-type group was not significant compared to the TP53 mutant group.

Reportedly, serum systemic inflammatory reaction (SIR) of tumor immune infiltration microenvironment is critical for regulating the malignant biological behavior of tumor cells in multiple solid tumors (28–31). According to a study, high NLR and PLR were associated with shorter OS in NSCLC with brain metastases (32). Mansfield et al. found that the PD-L1 expression and tumor-infiltrating lymphocytes (TILs) of paired primary lung cancers and brain metastases were significantly different; the PD-L1 expression and TILs were higher in lung cancer tissues than in brain metastases (33). Moreover, the study suggested that high NLR (≥4.95) had significantly more brain metastases at diagnosis than those with low NLR, particularly in the group with adenocarcinoma (34). However, the relationship between TILs and EGFR-TKIs in EGFR-mutant advanced LUAD with brain metastasis is unknown. Therefore, in the current study, the univariate and multivariate Cox analysis showed that the PLR and NLR ratio did not correlate with PFS and OS in advanced LUAD patients receiving EGFR-TKI therapy, while high NLR and PLR were associated with shorter time to brain metastasis. The results may indicate that SIR might release immune cytokines and inflammatory factors into the peripheral blood, activating the inflammatory immune response and promoting the tumor cells’ distant metastasis.

There were some limitations in this study. First, many studies showed that TP53/EGFR co-mutations were found in nearly 17%–70% of advanced NSCLC, while our study found TP53 and EGFR co-mutations in only 14.8% (35/236 patients), which had an insufficient sample size to analyze the effect of TP53-specific exons on intracranial metastasis in EGFR-mutated advanced LUAD patients receiving EGFR-TKI therapy. Second, there were only 48 patients with LBM, and the fact that this is a single-center study with a small sample size and a retrospective design might have induced selective bias. Many patients received first-, second-, or third-generation TKI drugs; those who were uniformly treated may lead to variable therapeutic effects on brain metastases.

In conclusion, TP53 wild type had a longer median OS than TP53 mutation in all LUAD or L858R patients. Moreover, the effect of median PFS and OS in the TP53 wild-type group was significantly longer than the TP53-mutant group in NBM patients. The TP53 wild-type group had a higher ORR and DCR than TP53 mutation with NBM, EBM, and LBM patients, regardless of the primary lung and brain metastatic lesions. Interestingly, it was found that TP53-mutated patients quickly progressed to brain metastasis. Therefore, it is imperative to clarify the predictive and prognostic TP53-mutated status in EGFR-mutant advanced LUAD patients with brain metastasis who underwent EGFR-TKI therapy and explore new molecular markers and treatment strategies for LUAD patients.

## Data availability statement

The original contributions presented in the study are included in the article/supplementary material. Further inquiries can be directed to the corresponding authors.

## Ethics statement

This study has been reviewed and approved by the ethics institution committee of First Affiliated Hospital of Nanchang University, Nanchang, China, all patients provided written informed consent and compliance with the declaration of Helsinki. All the patients’ data were kept confidential. The studies were conducted in accordance with the local legislation and institutional requirements. Written informed consent for participation was not required from the participants or the participants’ legal guardians/next of kin in accordance with the national legislation and institutional requirements.

## Author contributions

WG: Data curation, Formal analysis, Funding acquisition, Methodology, Resources, Software, Writing – original draft, Writing – review & editing. PL: Data curation, Formal analysis, Software. SW: Methodology, Validation, Writing – review & editing. JT: Conceptualization, Data curation, Writing – review & editing. JL: Methodology, Software, Writing – review & editing. JZ: Conceptualization, Data curation, Writing – review & editing. JX: Software, Writing – review & editing. JD: Investigation, Methodology, Writing – review & editing. FY: Data curation, Writing – review & editing. CS: Formal analysis, Software, Writing – review & editing. FQ: Methodology, Software, Writing – review & editing.
